# Food addiction and binge eating disorder are linked to shared and unique deficits in emotion regulation among female seeking bariatric surgery

**DOI:** 10.1186/s40337-023-00815-x

**Published:** 2023-06-13

**Authors:** Shahrzad Ahmadkaraji, Hojjatollah Farahani, Koosha Orfi, Fahimeh Fathali Lavasani

**Affiliations:** 1 Department of Clinical Psychology, School of Behavioral Sciences and Mental Health, Tehran Institute of Psychiatry, Iran University of Medical Sciences, Tehran, Iran; 2 Minimally Invasive Surgery Research Center, Rasool-e-Akram Hospital, Iran University of Medical Sciences, Tehran, Iran; 3 Department of Psychology, Tarbiat Modares University, Tehran, Iran; 4 Department of Clinical Psychology, University of Social Welfare and Rehabilitation Sciences, Tehran, Iran

**Keywords:** Food addiction, Binge eating disorder, Emotional eating, Emotion dysregulation, Bariatric surgery

## Abstract

**Background:**

Problematic eating behaviors can indicate obesity-related problems. Food addiction (FA) is not classified as an official diagnosis. However, given the many commonalities between FA and binge-eating disorder (BED) within the context of obesity, it is imperative to conduct a comparative investigation. The current study aimed to identify overlapping and distinctive features in emotion dysregulation as an underlying mechanism and emotional eating as a clinical feature among four groups of females with obesity seeking bariatric surgery.

**Methods:**

Data on emotion dysregulation and emotional eating were derived from the total 128 Females with obesity seeking bariatric surgery (*M*_age_ = 38.91 ± 10.59, *M*_BMI_ = 42.10 kg/m^2^ ± 4.43) divided into four groups: those with FA (*n* = 35), BED (*n* = 35), BED + FA (*n* = 31) and a control group of individuals with obesity only (OB; *n* = 27), using well-established measures.

**Results:**

Regarding descriptive statistics, the BED + FA group showed the highest levels of emotional dysregulation (M = 111.09) and emotional eating (M = 46.80), while the OB group acquired the lowest scores (M = 70.44 and M = 27.29, respectively). Univariate analyses of variance revealed significant differences between the four groups in terms of emotion dysregulation F(3, 124) = 24.63, *p* < .01 and emotional eating F(3, 124) = 26.26, *p* < .01. All of the emotion dysregulation domains revealed significant differences too. Pairwise comparisons using Bonferroni post hoc tests did not reveal any significant differences between BED + FA and BED groups, while all of our other hypotheses regarding this matter were confirmed.

**Conclusions:**

The study found that individuals with obesity and comorbid BED exhibit greater emotional dysregulation compared to those with OB or FA, indicating a need to assess BED in individuals with obesity. Emotion dysregulation may be linked to increased BED and FA, but those with BED seem more affected by limited access to emotion regulation strategies. These findings support the notion that PEBs are associated with emotion dysregulation and underscore the need for tailored interventions that target emotion regulation skills before and after bariatric surgery.

## Abstract (plain language summary)

BED and addictive-like eating are associated with wide degrees of psychopathology among individuals with obesity who are candidates for bariatric surgery and have several overlapping characteristics. Emotional dysregulation is a key mechanism in addiction disorders and BED, which may be obesity-maintenance factors. The current study examined overlapping and distinctive features of emotion dysregulation and emotional eating among 128 females with obesity who were seeking bariatric surgery. They were divided into four groups: FA, BED, BED + FA, and a healthy control group with OB. In general, female surgery candidates endorsing problematic eating behavior reported greater deficits in emotional regulation and emotional eating. Emotional dysregulation was more severe in those with obesity and comorbid BED compared to those in the OB group. These findings suggest that examining eating disorder symptomatology, especially a diagnosis of BED, is warranted in this population.

## Background

Obesity has been recognized as a major public health issue worldwide [1], and individuals with obesity who present for bariatric surgery often exhibit problematic eating behaviors (PEBs), including various eating disorders and addictive-like eating [2].

Examining the specific factors that contribute to obesity can help identify tailored treatment strategies prior to surgery, ultimately improving patients' outcomes after surgery [3].

Recent studies suggest that emotion dysregulation could have a crucial role in the onset and maintenance of obesity [4, 5]. Emotion regulation is a multifaceted construct comprising emotional awareness, acceptance, clarity, and the ability to engage in effective goal-directed behaviors. Emotion dysregulation occurs when an individual has deficiencies in any of these domains [6]. Research on emotional dysregulation emphasizes that eating is a maladaptive coping strategy for emotional distress [7]. Specifically, individuals with obesity have greater difficulty identifying and describing their emotions [8]. In addition, patients with obesity seeking bariatric surgery are often characterized by several PEBs, such as binge-eating disorder (BED) and food addiction (FA), which are characterized by emotion dysregulation [9, 10].

Even though it is not an officially recognized diagnosis, FA is a topic of ongoing scientific research and controversy [11]. Yale Food Addiction Scale 2.0 (YFAS 2.0) was created by using diagnostic criteria of substance use disorder (SUD) to assess symptoms of addictive-like eating [12]. For some individuals, palatable foods (e.g., processed foods) may have addictive potential and trigger the symptoms of eating problems, such as cravings and overeating [13]. FA has been related to eating disorders, particularly BED, which is related to obesity [14, 15]. BED is characterized by eating an excessive amount of food within a short period of time (at least once per week for three months) and experiencing a feeling of lack of control over eating. A lack of compensatory behaviors and significant distress are other hallmarks of BED [16].

BED is more common in females than males and in individuals with obesity (5% to 30%), especially those seeking obesity treatment [17], and FA has been observed in up to 35% of bariatric surgery patients [18]. Although studies show a large overlap between FA and BED, little is known regarding the distinction between BED and FA in individuals with obesity [19, 20].

Indeed, emotional dysregulation is a critical factor underpinning FA and BED [19, 21]. It has been postulated that problematic eating behaviors may be a coping strategy for extreme emotional states [22]. Emotion dysregulation is highly linked to addictive disorders and is now recognized as a central component of treatment for these disorders [23]. In the case of eating disorders, a recent meta-analysis revealed the transdiagnostic nature of emotion dysregulation [24]. Some evidence found that negative emotions trigger binge eating in BED patients but not in those with obesity without BED [25].

Numerous studies have explored the link between emotion regulation and emotional eating, characterized by excessive food intake in response to negative emotions [26, 27]. Emotional eating is prevalent among bariatric surgery candidates and is associated with poorer surgical outcomes [28].

Emotional eating is strongly associated with binge eating severity [29], and individuals with FA score higher on the Emotional Eating subscale of the Dutch Eating Behavior Questionnaire (DEBQ-E) than non-affected individuals, suggesting that emotional states may play an important role in triggering addictive-like eating behaviors [30].

Several studies conducted among treatment-seeking individuals with obesity have suggested that, alongside the possibility of BED and FA as independent clinical entities, their comorbidity implies a more severe subtype of BED [31, 32].

A comprehensive understanding of the distinction between BED and FA by investigating possible shared and unique underlying factors (e.g., emotion dysregulation) and clinical features (e.g., emotional eating) can have clinical importance and inform tailored treatment approaches for individuals with obesity [20].

The primary objective of this study was to assess deficits in emotion regulation and emotional eating associated with BED and FA. We evaluated four groups of females with obesity seeking bariatric surgery: those with FA, BED, BED + FA, and a healthy control group with obesity (OB) matched for age, sex, and BMI. Drawing upon previous literature [30, 31], we formulated the following hypotheses: (1) Patients in the BED + FA group have more emotional eating and emotional dysregulation than those in the OB, FA, and BED groups; (2) Patients in the FA group have poorer emotional regulation than the OB groups; (3) Patients in the BED group have greater difficulties in emotion regulation than the OB group. Due to the dearth of data, no evidence-based hypothesis was derived about potential variations in emotion dysregulation between FA and BED participants.

## Methods

### Participants and procedure

One hundred twenty-eight patients, aged 18 to 65 (*Mean* = 38.91, *SD* = 10.59), were recruited from patients seeking bariatric surgery and undergoing pre-surgical psychological evaluation at The Minimally Invasive Surgery Research Center of Iran University of Medical Sciences.

Inclusion criteria were: (1) confirmation that individuals were considering bariatric surgery following evaluation by medical professionals, and (2) obesity grade 2 (BMI ≥ 35 kg/m^2^) and at least one obesity-related disease (e.g., type 2 diabetes) or obesity grade 3 (BMI ≥ 40 kg/m^2^). The exclusion criteria adhered to guidelines for bariatric surgery and excluded individuals with untreated severe mental disorders, such as psychotic disorders and past substance-use disorders. These exclusions aimed to prevent disruptions to emotional regulation assessments. BED was diagnosed using the Eating Disorder Examination (EDE) [33], and to determine the participants' eligibility for inclusion as having FA or not, the YFAS 2.0 was used. Based on scores from the YFAS 2.0 and the semi-structured interview, participants were divided into four groups: (1) Participants with comorbid BED and FA, (2) Participants who just met the criteria of BED, (3) Participants who just met the criteria of FA, (4) Participants who met the criteria for obesity but did not meet the criteria for BED or FA (control group). Individuals with clinically severe purging behaviors were excluded.

The Minimally Invasive Surgery Research Center of Iran University of Medical Sciences approved the current study. Subjects voluntarily participated in the study, and written informed consent was obtained. The patients completed the surveys before the preoperative psychological evaluation. The sampling spanned from June 2021 to December 2021.

### Measures

#### The Yale food addiction scale 2.0 (YFAS 2.0)

The Yale Food Addiction Scale 2.0 (YFAS 2.0) [12] is a revised version of the original Yale Food Addiction Scale (YFAS). The YFAS 2.0 is a self-report questionnaire designed to assess addictive-like eating behaviors. It consists of 35 items that measure symptoms of FA based on the diagnostic criteria for SUD in the Diagnostic and Statistical Manual of Mental Disorders, 5th edition guidelines (DSM-5) [16]. The YFAS 2.0 rates symptoms on a scale of 0 to 11, with scores indicating the level of "severity" of FA. Scores can range from no FA (1 or fewer symptoms and no clinical significance) to mild (2 or 3 symptoms with clinical significance), moderate (4 or 5 symptoms with clinical significance), or severe FA (6 or more symptoms with clinical significance). The YFAS 2.0 has shown good psychometric properties in bariatric surgery patients [34]. In the present study, we used the validated Persian version [35], and Cronbach's alpha for the YFAS 2.0 was 0.95.

#### Eating disorder examination (EDE)

BED was diagnosed using a shorter version of the Eating Disorder Examination (EDE) to evaluate if participants had BED (as per the DSM-5). Those who reported experiencing one or more episodes of binge eating per week for the past three months and also met other associated behavioral criteria were identified as having BED. The EDE is a well-established, investigator-based interview method that has been widely utilized for assessing various forms of eating disorder psychopathology [33]. It has been shown to be a reliable instrument [36].

#### The binge eating scale (BES)

The Binge Eating Scale (BES) [37] is a self-report questionnaire composed of 16 items, which evaluates the severity of binge eating episodes and their related feelings and thoughts. The total score on the BES ranges from 0 to 46, with higher scores indicating more severe binge eating. To classify individuals based on the severity of their binge eating, we employed a widely-used categorization system: absence of binge eating (scoring ≤ 17), mild to moderate binge eating (scoring 18–26), and severe binge eating (scoring ≥ 27) [38]. In this study, we used the validated Persian version of the BES [39], and Cronbach's alpha for this sample was calculated to be 0.88.

#### The difficulties in emotion regulation scale (DERS)

The Difficulties in Emotion Regulation Scale (DERS) is a self-report questionnaire to evaluate difficulties with emotion regulation [6]. This measure consists of 36 items that are responded to using a 5-point Likert scale, with scores ranging from 1 to 5 indicating "almost never" and "almost always," respectively. It assesses six dimensions of emotion dysregulation, including lack of awareness of emotional responses (awareness), non-acceptance of emotional responses (non-acceptance), lack of clarity of emotional responses (clarity), limited access to emotion regulation strategies (strategies), impulse control difficulties (impulse), and difficulties engaging in goal-directed behaviors (goals). In the present study, we used the validated Persian version, and the internal consistency of the overall DERS score was found to be high (α = 0.94). The subscales exhibited adequate reliability (awareness = 0.74, non-acceptance = 0.87, clarity = 0.82, strategies = 0.86, impulse = 0.87, and goals = 0.83).

#### The dutch eating behavior questionnaire-emotional eating (DEBQ-E)

The Persian version of the DEBQ-E assessed participants' emotional eating behavior. The DEBQ-E consists of 13 items rated using a Likert scale with responses ranging from 1 to 5 [40]. In the present study, Cronbach's alpha of the DEBQ-E score was 0.88.

### Statistical analyses

For our statistical analysis, we utilized IBM SPSS Statistics version 22. After determining groups’ characteristics, we conducted two chi square tests of independence to determine whether observed differences in the frequency of BED and FA cases are significant. Then, we conducted two-tailed tests with a significance level of *(p* < 0.05*)*. Our study focused on the emotion dysregulation subscales rather than the total score. To compare between groups, we used univariate analyses of variance (ANOVAs) for continuous variables. The study groups were matched according to age and BMI. Before conducting these analyses, we assessed the homogeneity of variances using the Levene test and checked for normality assumptions using kurtosis and skewness. The variable was considered normally distributed if skewness was less than 3 and kurtosis was less than 10. All variables except BMI were normally distributed and demonstrated homogeneous variances. In addition, the Bonferroni test was used for pairwise comparison, and estimated omega squared was used to determine the effect sizes. The interpretation of omega squared effect size followed the general guidelines proposed by Stevens [41]. Specifically, effect sizes of 0.01, 0.06, and 0.14 were considered as small, medium, and large effects, respectively.

## Results

### Demographic variables

Among a group of patients with obesity seeking bariatric surgery, 35 participants met the criteria for BED, and another 35 met the criteria for FA. Additionally, 31 participants fulfilled BED and FA criteria, while 27 individuals were classified under the OB group. After performing independent t-tests on all four groups, no significant differences in age and BMI were found. Consequently, the groups were matched based on these variables (Table 1).

**Table 1:** Groups' characteristics

Characteristics	BED + FA n = 31	BED n = 35	FA n = 35	OB n = 27	Test statistics		
	*M (SD)*	*M (SD)*	*M (SD)*	*M (SD)*	*F*	*df*	*p*
Age	37.19 (9.02)	37.85 (11.4)	40.02 (10.60)	40.81 (11.26)	0.8	3	0.494
BMI	43.04 (3.45)	41.72 (4.90)	41.76 (3.62)	41.96 (5.67)	0.617	3	0.605

	*n*	*n*	*n*	*n*	*X*^*2*^	*df*	*p*
FA severity (mild/moderate/severe)	2/8/21	–	11/13/11	–	10.342	2	0.006
BE severity (mild to moderate/severe)	9/22	16/19	–	–	1.944	1	0.163

*M* mean, *SD* standard deviation, *DF* degrees of freedom, *FA* food addiction, *BED* binge-eating disorder, *OB* Obesity only, *BMI* body mass index

Based on the results of a chi-square test, there was a significant difference in the frequency of severe FA cases between the two groups, χ^2^(1) = 9.86, *p* < 0.01. More specifically, in the BED + FA group, 21 individuals (67.7%) were classified as severe FA, while in the FA group, 11 individuals (31.4%) were classified as severe FA. These findings suggest that the frequency of severe cases differs significantly between these two groups.

Regarding severe BED, there was no significant difference in the frequency of severe BED cases between the BED group and BED + FA group, χ^2^(1) = 0.93, *p* = 0.33. Specifically, in the BED + FA, 22 individuals (71.0%) were classified as severe BED, while in the BED group, 19 individuals (54.3%) were classified as severe BED. These findings suggest that there is no evidence to support a significant difference in the frequency of BED cases between the two groups.

### Descriptive statistics and comparisons between groups

As indicated in Table 2, the BED + FA group had the highest mean scores in all emotion dysregulation domains and emotional eating compared to the other groups. Conversely, the OB group had the lowest mean scores (Table 2). Therefore, a series of ANOVAs demonstrated significant differences with moderate to large effect sizes in emotion dysregulation domains and emotional eating among the four groups. Subsequently, pairwise comparisons revealed that the BED + FA, BED, and FA groups had significantly higher scores than the OB group in non-acceptance, goals, impulse, and strategies domains of emotional dysregulation, as well as in emotional eating. Similarly, the BED + FA and BED group exhibited substantially higher scores in awareness and clarity than the OB group.

**Table 2:** Comparison of the clinical characteristics of patients in the study based on their group allocation

Variables		BED + FA	BED	FA	OB	ANOVA			
		M (SD)	M (SD)	M (SD)	M (SD)	*F*	*p*	*ω*^*2*^	Post hoc
	Total score	111.09 (26.92)	106.82 (18.82)	91.88 (17.06)	70.44 (14.70)	24.63	< 0.001	0.35	1,2,3 > 4; 1,2 > 3
DERS domains	Non-acceptance	20.41 (6.61)	19.37 (5.80)	16.60 (5.15)	11.22 (3.12)	16.72	< 0.001	0.27	1,2,3 > 4; 1 > 3
	Goals	17.93 (4.18)	16.94 (4.09)	14.62 (3.86)	11.37 (4.24)	14.79	< 0.001	0.24	1,2,3 > 4; 1 > 3
	Impulse	18.45 (5.75)	17.65 (4.73)	15.05 (4.22)	10.92 (3.99)	14.85	< 0.001	0.24	1,2,3 > 4; 1 > 3
	Awareness	16.93 (4.73)	16.48 (3.95)	14.40 (3.58)	12.92 (3.68)	6.47	< 0.001	0.11	1,2 > 4
	Strategies	24.77 (8.12)	24.6 (6.92)	20.28 (5.79)	15.03 (4.88)	14.21	< 0.001	0.23	1,2,3 > 4; 1,2 > 3
	Clarity	12.58 (4.10)	11.77 (4.59)	10.91 (2.31)	8.96 (3.20)	5.13	0.002	0.09	1,2 > 4
DEBQ	Emotional eating	46.80 (8.57)	42.14 (8.86)	40.45 (7.55)	27.29 (10.57)	25.26	< 0.001	0.36	1,2,3 > 4; 1 > 3

*M* mean, *SD* standard deviation, *ω*^*2*^ estimated omega squared, *DERS* Difficulties in Emotion Regulation Scale; *DEBQ* Dutch Eating Behavior Questionnaire

## Discussion

The increasing evidence of comorbidities between BED and FA indicates the presence of more psychopathological symptoms and poorer bariatric surgery outcomes. This highlights the importance of investigating potential factors underlying these conditions. Therefore, this study aimed to investigate the implications of FA on obesity in patients with and without BED seeking bariatric surgery. A group with obesity without BED and FA was included as a control group. Our main finding, which builds upon earlier research, is that individuals with the comorbidity of FA and BED tend to exhibit greater emotional dysregulation and emotional eating compared to those without FA. Another finding revealed that the OB group was more emotionally regulated and had less emotional eating than the BED + FA, BED, and FA groups. Overall, these findings are similar to those reported in people with severe obesity but without any PEBs [20, 25, 42] and support our hypotheses suggesting that the presence of different forms of PEBs seems to represent important subtypes of patients with obesity and are associated with more failure of emotional regulation and higher emotional eating. In this section, we will discuss the findings in detail.

This study revealed that patients in the BED group exhibited greater deficits in all domains of emotion dysregulation compared to those in the OB group. According to the affect regulation model [43], binge eating is a maladaptive way to alleviate negative emotions through food consumption. Our findings align with those of Conti et al. [44] and Benzerouk et al. [45], who found that Patients with BED reported significantly higher emotional dysregulation than those with obesity but without BED.

In comparison to the OB group, individuals who only met the criteria for FA had significantly worse deficits in most emotion regulation domains, including acceptance of emotions, goal-directed behavior, impulsive control behaviors, and access to emotion regulation strategies. Similarly, Hardy et al. [46] showed that individuals with FA had considerable impairments in all of the emotion regulation domains except emotional awareness. These findings align with recent research showing that people with FA may have a heightened awareness of their feelings but lack the emotional regulation abilities necessary to cope with high levels of negative emotions [42, 46]. This aligns with studies showing that pre-surgical FA is associated with a wide range of psychological disturbances [2].

In this study, patients with BED and BED + FA did not differ in emotional dysregulation domains or emotional eating. This finding is consistent with the study of Ivezaj et al. [20], which did not show elevated clinical disturbance in the comorbid BED + FA compared to BED without FA. However, this contrasts with the study of Gearhardt et al. [31], which showed that comorbidity of FA and BED was associated with higher deficits in emotion regulation and disordered eating. This difference may be due to the remarkably higher rate of BMI (BMI ≥ 40) in our sample than in the study mentioned above (BMI ≥ 30). In addition, compared to the FA group, the BED + FA group exhibited greater deficits in emotion dysregulation, more severe emotional eating, and more severe cases of FA than those with only FA. Consistent with previous studies [47], these results imply that the observed differences relate to more severe symptoms in the case of comorbidity. These findings support our hypothesis that patients in the BED + FA group have more emotional eating and emotional dysregulation than those in the OB and FA groups.

The findings also indicate that FA and BED are associated with shared and distinct emotion regulation difficulties. It appeared that the only significant distinction between BED and FA was limited access to emotional regulation strategies, for which the BED group had greater difficulties in emotion regulation. This confirms the results reported by Benzerouk et al. [45], which showed that BED patients were more prone to report limited access to emotion regulation strategies [48]. Moreover, BED and FA share a common clinical feature, including loss of control [49]. Uncontrolled eating is most accurately described by the construct of emotional eating, which attempts to employ eating as a coping mechanism when negative emotions arise [50]. Previous research has demonstrated that individuals with comorbid SUD and ED are more susceptible to experiencing intense urges to eat when dealing with emotional distress and may be at an increased risk of engaging in emotional eating [51]. These findings support our original hypothesis.

However, the mechanisms underpinning this observation are not yet known. Therefore, this finding needs to be confirmed in larger, independent groups of people with FA and BED. These findings are consistent with the growing body of evidence indicating that emotion regulation difficulties are likely intrinsic to numerous forms of psychopathology. We also added to the existing literature by clarifying the underlying mechanisms involved in each subtype of PEBs [52].

### Strength and limits

To the best of our knowledge, this study is the first to explore FA and BED regarding underlying factors and clinical features in a sample of patients with obesity seeking bariatric surgery. Including two distinct groups of patients with BED and FA allows us to investigate the unique contribution of PEBs to obesity psychopathology and differentiate the clinical relevance of characteristics shared in both conditions. However, the results of this study must be interpreted based on some limitations. Assessments mainly relied on self-reports, which are susceptible to response or desirability bias. However, we utilized validated evaluation instruments (e.g., the YFAS 2.0, BES). This study only included females, which limited its generalizability. Also, given the cross-sectional nature of our design, we did not conduct a follow-up study to investigate the relationship between preoperative PEB-related psychopathologies and postoperative weight loss, which warrants further investigations.

### Implications

Emotional dysregulation domains seem crucial in obesity and eating disorder psychopathologies. The findings in this study help to differentiate which emotional dysfunctions are related to obesity as a whole and which are specific to PEBs [45, 53].

Overall, our results lead us to believe that improving emotional skills would ameliorate the patients' approach to food, which could significantly improve their eating behaviors before surgery. It could also create a more favourable outcome following bariatric surgery [54].

## Conclusion

The pattern of results showed that both common and disorder-specific deficiencies in emotion regulation are associated with BED and FA. The current study identified greater emotional dysregulation in individuals with obesity and comorbid BED compared to those with OB or FA. Therefore, the assessment of eating disorders, specifically a diagnosis of BED, is suggested in individuals with obesity. Overall, emotion dysregulation may be linked to increased binge eating and FA. However, those with BED may be more affected by limited access to emotion regulation strategies. By considering the aspects related to PEBs in distinct groups of FA and BED, this study indicates the importance of evaluating the facets of emotional dysregulation involved in obesity psychopathology.

## Data Availability

The datasets analyzed during the current study are available from the corresponding author upon reasonable request.
