# Correction: Food addiction and binge eating disorder are linked to shared and unique deficits in emotion regulation among female seeking bariatric surgery

**DOI:** 10.1186/s40337-024-00989-y

**Published:** 2024-02-15

**Authors:** Shahrzad Ahmadkaraji, Hojjatollah Farahani, Koosha Orf, Fahimeh Fathali Lavasani

**Affiliations:** 1 Department of Clinical Psychology, School of Behavioral Sciences and Mental Health, Tehran Institute of Psychiatry, Iran University of Medical Sciences, Tehran, Iran; 2 Minimally Invasive Surgery Research Center, Rasool-E-Akram Hospital, Iran University of Medical Sciences, Tehran, Iran; 3 Department of Psychology, Tarbiat Modares University, Tehran, Iran; 4 Department of Clinical Psychology, University of Social Welfare and Rehabilitation Sciences, Tehran, Iran

**Correction: Journal of Eating Disorders(2023)11:97** 10.1186/s40337-023-00815-x

The original article [1] has incomplete funding information. The sponsorship from Takeda Pharmaceutical Australia to cover the Article Processing Charge was not mentioned in the Funding note. The correct Funding note is as follows:

**Funding**

The article has received sponsorship from Takeda Pharmaceutical Australia to cover the Article Processing Charge.

The original article [1] has been corrected.
